# Signaling by Type I Interferons in Immune Cells: Disease Consequences

**DOI:** 10.3390/cancers16081600

**Published:** 2024-04-22

**Authors:** Markella Zannikou, Eleanor N. Fish, Leonidas C. Platanias

**Affiliations:** 1 Robert H. Lurie Comprehensive Cancer Center, Division of Hematology-Oncology, Feinberg School of Medicine, Northwestern University, 303 East Superior Ave., Chicago, IL 60611, USA; 2 Toronto General Hospital Research Institute, University Health Network, 67 College Street, Toronto, ON M5G 2M1, Canada; en.fish@utoronto.ca; 3 Department of Immunology, University of Toronto, 1 King’s College Circle, Toronto, ON M5S 1A8, Canada; 4 Department of Medicine, Jesse Brown Veterans Affairs Medical Center, 820 S. Damen Ave., Chicago, IL 60612, USA

**Keywords:** interferons, signaling, immune cells, cancer, dendritic cell, macrophage, NK cells, neutrophils, T cell, B cells, tumor microenvironment

## Abstract

**Simple Summary:**

IFNs are cytokines that play critical roles in the immune defense mechanisms that prevent virus spread. They also exhibit regulatory effects on the immune system and contribute to the elimination of malignant cells. These cytokines mediate their effects by binding on unique receptors on the surface of immune cells, initiating signaling cascades that trigger expression of IFN-stimulated genes (ISGs) that ultimately drive expression of specific proteins that mediate the various interferon responses and effects. In this review, we discuss the mechanisms by which interferons control different types of cells of the immune system, as well as malignant cells to mediate their important biological properties.

**Abstract:**

This review addresses interferon (IFN) signaling in immune cells and the tumor microenvironment (TME) and examines how this affects cancer progression. The data reveal that IFNs exert dual roles in cancers, dependent on the TME, exhibiting both anti-tumor activity and promoting cancer progression. We discuss the abnormal IFN signaling induced by cancerous cells that alters immune responses to permit their survival and proliferation.

## 1. Introduction

By preventing the spread of viruses, IFNs are essential in the immune defense process [1,2]. According to the immunosurveillance hypothesis proposed by Thomas and Burnet, IFNs are important constituents of the immune system that detect and eliminate cancerous cells [3,4]. In 1994, Schreiber and colleagues provided initial experimental evidence of immunosurveillance, by highlighting the crucial role of IFNγ signaling in cancer immunoediting [5,6,7]. IFNs have various activities, which have been implicated in eliminating virus infections, promoting autoimmune disorders and metabolic syndromes, and clearing cancers [8]. IFNs are classified into three Types: I, II, and III. The defining factors for their classification include the type of cell that produces the IFN, the molecular structure of the IFN itself, the cell surface receptor that recognizes the IFN, the specific associated signaling pathways involved, and the particular responses that the IFN triggers. By considering all these factors, we can unravel the complex mechanisms that govern cellular responses to different IFNs in the appropriate contexts.

The Type I IFNs (IFN-I) have been studied most extensively [4]. The human genome contains 17 distinct IFN-I consisting of 13 subgroups of partially homologous IFN-αs (approximately 70–80% identity), in addition to IFN-β, IFNε, IFNκ, and IFNω, which have lesser homology (30–50%) [9,10]. All nucleated cells produce IFN-I subtypes that bind to the ubiquitously expressed IFNα/β receptor (IFNAR), triggering the expression of hundreds to thousands of IFN sensitive genes (ISGs) [11,12,13,14]. IFNAR is a heterodimeric receptor located on the cell surface (IFNAR1 and IFNAR2) [15]. IFNAR signaling leads to activation of JAK-STAT pathways [16,17] and non-canonical signaling pathways [18].

The single Type II IFN (IFN-II) is IFNγ. IFNγ is primarily secreted by NK, NKT, CD4 Th1, and cytotoxic effector cells [19] and activates the IFNGR receptor (IFNGR1 and IFNGR2), expressed on nearly every cell type (e.g., astrocytes, microglia, and oligodendrocytes) [20,21,22], except mature erythrocytes [23]. More recently, the Type III IFNs (IFN-III) were identified (IFN-λ1,2,3 and λ4) [24]. IFN-III exhibits a structural similarity to IFNγ, while their functional characteristics more closely resemble (IFNsα/β) [25]. Epithelial and immune cells are selectively targeted by the IFNλs, mediated by a receptor complex comprising IL-10R2 and IFNLR1 [14].

IFNs exert their pleiotropic effects in response to challenges to the host immune system. These include limiting the propagation of viruses, mediated in part by stimulating natural killer (NK) cell cytotoxicity, promoting maturation of antigen-presenting cells (APCs), facilitating the clonal expansion and survival of virus-specific T cells (CD4 and CD8), enhancing B cell activation, and inducing cell death. The literature is replete with data that have revealed the critical role IFN-I have in clearing virus infections and certain malignancies [26,27,28,29]. Cells produce IFN-I when pattern recognition receptors (PRRs) detect pathogen-associated molecular pattern molecules (PAMPs) and damage-associated molecular patterns (DAMPs). In addition to PAMPs and DAMPs, CSF1, RANK, and estrogens can induce IFN-I [30]. Changes in lipid metabolic pathways and IFN-I production require a nutrient detector, the mammalian target of the rapamycin (mTOR) [31]. Biosynthesis and cellular metabolism are strongly influenced by IFN-I production [32,33]. A basal systemic IFN response from the commensal microbiota regulates innate immune responses and maintains homeostasis [34,35,36].

## 2. Cancer Cells

**Figure 1:**
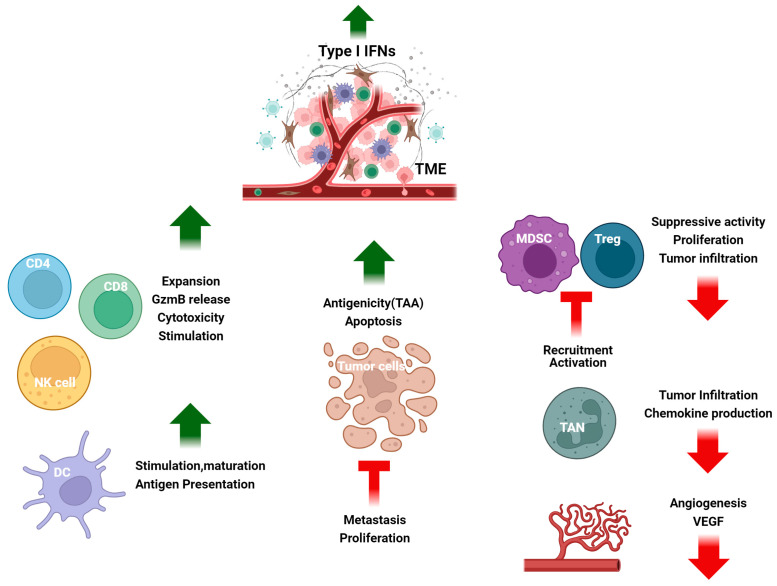
The tumor microenvironment (TME) is affected by IFN-I. IFN-I stimulate antigen-dependent maturation of DCs, the expansion and cytotoxicity of CD4^+^ and CD8^+^ T cells and NK cells. IFN-I enhance the production of *co-stimulatory* molecules, activating the STAT3-Granzyme B pathway and thereby limiting the immunosuppressive TME, by inhibiting Tregs, MDSCs, and angiogenesis and converting tumor-associated neutrophils into antitumor neutrophils. Overall, IFN-I promote antitumor immunity and inhibit tumor progression and metastasis. DC—dendritic cell, NK—natural killer, VEGF—vascular endothelial growth factor, Neu—neutrophils, Treg—T regulatory T cells, MDSC—myeloid derived suppressor cell. Created with https://BioRender.com, accessed on 9 March 2024.

A subset of ISGs has been identified in different malignancies, associated with DNA damage resistance, that is predictive of responsiveness to IR and chemotherapy [37]. Notably, this ISG signature closely resembles the ISG subset activated by U-ISGF3 during an antiviral response [38]. Cancer cells can maintain the expression of proteins driven by U-ISGF3. IFN-I are present in low concentrations in both the TME and within tumors, promote the sustained expression of this IFN-related DNA damage resistance subset of ISGs, and their protein products offer significant protection against DNA damage [38,39]. However, acute exposure to high levels of IFN-I, whether administered as therapy or in response to severe DNA damage caused by ionizing radiation, is cytotoxic [40]. In certain types of tumors, IFN-I can inhibit the epithelial-mesenchymal transition (EMT), whereas oncostatin M can have the opposite effect, promoting a pro-tumor, stem-like, mesenchymal phenotype [41]. IFN-I stimulate the antigen dependent maturation of DCs and the expansion and cytotoxicity of NK, T, and B cells to enhance the body’s immune response against cancer. In detail, IFN-I signaling stimulates DCs to deliver antigens to CD8^+^ T-cells by increasing the expression of MHC-I, MHC-II, CD40, and other molecules [42,43]. CD8 T cells, assisted by CD4^+^ T cells, can trigger the STAT3-Granzyme B (GzmB) pathway to eliminate tumor cells. IFN-I counteract the development of an immunosuppressive tumor microenvironment by reducing Treg uptake and activation. IFN-I suppress MDSCs and stimulate M1-polarized proinflammatory macrophages, producing proinflammatory cytokines (e.g., TNF-α, IL-1, IL-6, IL-8, IL-12, and IL-18) [44,45,46]. IFN-I help transform tumor-associated neutrophils into an antitumor state, improving the overall picture/frame (Figure 1).

MNK kinases, activated downstream of IFN-induced MAP kinase pathways, enable mRNA translation of ISGs in immune and malignant cells [47,48,49,50,51]. ULK1, the mammalian homolog of the UNC-51 kinase of *Caenorhabditis elegans*, is necessary for activating p38 MAPK and the transcription of ISGs involved in anti-cancer effects and responses [52,53,54]. The presence of ULK1 is critical for the regulation of immune antitumor responses, as its absence decreases IFNγ-dependent gene expression involved in inflammation regulation [55]. A recent study of immune infiltration analysis by CIBERSORT algorithms revealed that in diabetic nephropathy, ULK1 had a positive correlation with neutrophils and a negative correlation with M1 and M2 macrophages [56], further supporting the importance of ULK1 in regulating an inflammatory response.

Schlafen (SLFN) proteins play an essential role in regulating various cellular functions associated with cell growth and differentiation, immune cell growth and maturation, and antiviral activity [57,58,59]. SLFNs are widely expressed in mammals [57,60,61,62,63,64,65,66] and specific human SLFNs have been associated with pathological conditions. Specifically, recent research has shown a correlation between the presence of SLFN5 and an increased risk of gastric cancer in patients with metaplasia [67] On the other hand, changes in SLFN14 have been associated with a disorder characterized by severe thrombocytopenia and abnormal platelet production, leading to excessive bleeding [68]. The immunodeficiency condition [68] that resembles the Elektra phenotype in mice [69] has also been connected to a single copy deletion of the human SLFN gene locus region on chromosome 17q12. SLFN proteins can stimulate IFN responses with antiproliferative/antineoplastic effects and antiviral responses; however, they can also delay antitumor and antiviral responses [57,62,65,66,70]. Elevated levels of SLFN11 are strongly associated with ISG expression in both breast cancer [63] and small-cell lung cancer [71]. In the case of glioblastoma multiforme, SLFN5 can inhibit Stat1 transcriptional activity and suppress ISG expression, underlying its tumorigenic effects [72]. Similarly, mouse Slfn2 suppresses IFN-dependent genes, and knocking out the Slfn2 gene leads to increased IFN-inducible antiviral responses because of a decrease in NF-κB activation resulting from the interaction between Slfn2 and PPP6R1 [60]. SLFN11 may be involved in an IFN-I response to HIV infection, as it is IFN-inducible and possesses anti-HIV1 characteristics [73].

IFN-I have a role in chromatin remodeling and gene expression reprogramming [74]. IFN-I have been associated with triggering the histone demethylase, KDM1B, to promote transcriptional reprogramming of cancer cells towards cancer cell stemness and immune evasion [75,76,77].

## 3. Dendritic Cells (DCs)

IFN-I effectively trigger innate and adaptive immune responses, leading to both direct inhibition of tumor cells and indirect immune-cell mediated antitumor effects. This is achieved in part by stimulating the development and stimulation of DCs and macrophages for antigen processing, amplifying the secretion of granzymes and perforin by both CD8^+^effector T lymphocytes and NK cells [78,79], and boosting the proportion of memory T lymphocytes [80,81,82]. Many IFN-stimulated genes (ISGs) are activated in response to engagement of the IFN-activated JAK-STAT pathway and other signaling pathways that mediate responses [44,83,84,85].

Type I IFNs affect DC maturation, differentiation, and activity [44]. In the presence of IFN-I, there is a reduction in endosomal–lysosomal acidification, which in turn promotes the retention of cell-associated antigens within RAB5+ and RAB11+ compartments [86]. IFN-I activate CCR7 cell surface expression, which in turn enables the migration of antigen-bearing DCs to the lymph nodes to interact with and activate T cells. DCs elicit immune responses against tumors by presenting tumor-associated antigens to CD8^+^ T cells. IFN-I prolong the life span of antigen-bearing DCs, for the induction of effective immune responses, by upregulating Bcl-2 and Bcl-xL [86,87]. Studies have demonstrated that IFN-I signaling enhance DC capacity to activate tumor-specific CD8^+^ T cells. This is accomplished by DCs capturing antigenic particles from apoptotic cells and upregulating the expression of co-stimulatory molecules such as MHC-I, MHC-II, CD40, CD80, and CD86 [42,86]. Mice that lack IFNAR1 receptors on their DCs cannot reject highly immunogenic tumor cells, and the DC capacity to for antigen cross-presentation is impaired [88].

**Figure 2:**
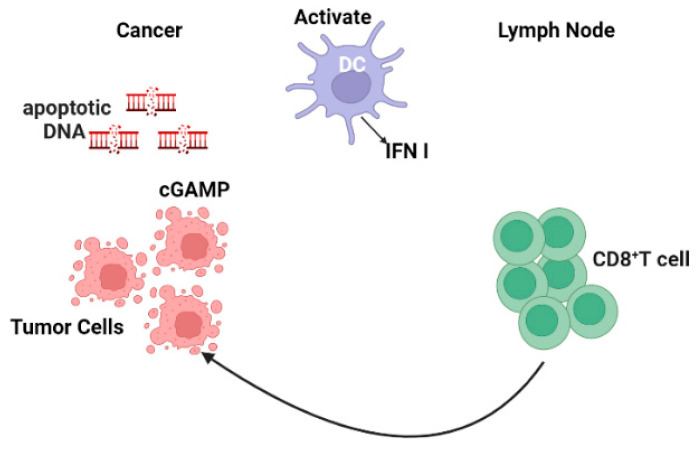
The STING pathway as a mechanism for tumor suppression. DNA damage-induced cGAS stimulates cytoplasmic DNA, activates STING which leads to an increase in IFN-I gene expression. IFN-I promote T cell migration to lymph nodes and activate DCs. Created with https://BioRender.com, accessed on 26 November 2023.

Numerous studies have documented the process whereby tumor cells release their DNA into the cytoplasm and are detected by the cGAS receptor, which then triggers the production of the messenger molecule, cGAMP [89,90,91]. Following activation, cGAMP interacts with STING, an adaptor protein located in the endoplasmic reticulum (Figure 2) [92]. The conformational changes generated by this interaction cause STING to relocate from the ER to perinuclear regions, and consequently activate TANK-binding kinase 1 (TBK1). Subsequently, TBK1, IFN regulatory factor3 (IRF3), IRF5, and IRF7 undergo phosphorylation. These activated factors then translocate to the nucleus where they interact with NF-κB to stimulate IFN-I gene expression [93,94]. Notably, unphosphorylated STAT2 inhibits STING-induced IFN-I production [41]. A recent study provided evidence that Mn2^+^ substantially impacts the development of antigen presenting cells (APCs) and their antigen presentation [95]. Mn2^+^ enhances the activation of effector cells to increase the subset of CD44*^hi^*CD8^+^ T cells. These effects are cGAS-STING pathway-dependent. Mn2^+^ administration significantly affects the immune response against tumors in different mouse models [95].

The production of CXCL10 by migratory CD8^+^ T cells in response to local IFN-I within the tumor microenvironment (TME) allows for the migration of effector T cells into the tumor [96,97,98]. Conventional DCs, when activated by IFN-I, migrate to lymph nodes, where they present tumor antigens to prime CD8^+^ T cell responses [98,99]. Notably, in certain TMEs, IFN-I exhibit opposing effects to induce the expression of cell death ligand 1 (PD-L1), enzyme IDO (indoleamine 2,3-dioxygenase), interleukin-10 (IL-10), regulatory T cells (Tregs) and other anti-inflammatory mediators [100], to evade the tumor-induced immune suppressive TME [101,102].

## 4. Natural Killer Cells

NK cells arise from NK lineage-restricted progenitors (NKPs) from common lymphoid progenitors (CLPs) [103,104]. Human NK cells, specifically the CD56^bright^CD16^−^ subset, exhibit enhanced responsiveness to inflammatory cytokines. IFNs and IL-2, IL-12, and IL-15 activate JAK-STAT signaling in NK cells to promote their growth, development, and functional responses [104,105]. IL2Rβ expression by NK precursors (NKPs) is essential for IL-15 activation of JAK1/3 and STAT5 and NK cell development [106,107,108,109]. Because of its function in IFN and IL-10 signaling, JAK1 plays a key role in NK cell biology [110]. In addition, JAK engages with the IL-4 receptor family (type 2 immune reactions), as well as the interleukin-6 (IL-6) family cytokines [110]. It is clear that JAKs and STAT5 play an essential role in NK cell development.

**Figure 3:**
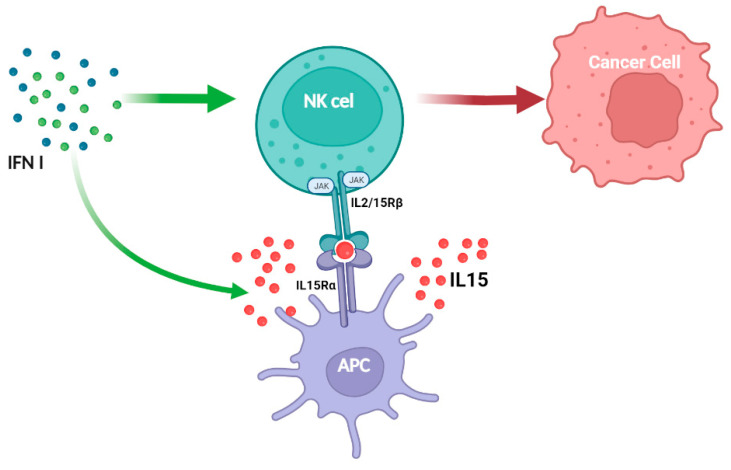
Type I IFNs augment NK cell antitumor effects directly and indirectly, in part mediated by APCs, which enhance the expression of IL-15/IL-15Rα complexes. Created https://BioRender.com, accessed on 26 November 2023.

JAK1/3 and STAT1/3/5 are involved in the IL-2 signal pathway in both T and NK cells, but unlike what is seen in T cells, IL-2 activates JAK2 and STAT4 in NK cells [111]. JAK2 and TYK2 transduce signaling for IL-12 family members and several STATS are activated in response to IL-12 [112]. Tyk2 association with IFNAR1, IL-10Rβ, IL-12Rβ1, and IL-13Rα1 mediates signal transduction for several cytokines (IFN-I, IL-10, and IL-12 family) [112]. IL-27 primarily signals through STAT1 and STAT3, whereas IL-23 primarily activates STAT3 and STAT4 in NK cells [113]. JAK2 activity supports developing DCs [114], which are responsible not only for producing IL-15 but also for the proper priming of NK cells [115]. Thus, JAK2 inhibition or deletion limits NK cell priming through DCs. Consistent with this, IL-15 therapy can prevent tumor dissemination caused by JAK2 inhibitors [116]. IFN-I enhance NK cell responses through direct and indirect action via DCs, causing IL-15 to promote NK cell activation and tumor elimination (Figure 3). Recently, STAT1 was shown to be involved in the immunological synapse of NK cells [117]. This non-canonical function regulates tumor surveillance and cytotoxicity. Notably, in primary mouse NK cells, CDK8 constitutively phosphorylates STAT1 on serine727, limiting NK cell cytotoxicity and tumor surveillance [118]. NK cells lacking STAT1 exhibit significant deficiencies in development, IFN-γ production, lytic granules secretion and memory [119,120,121]. Tumor-derived IFN favors the expression of PDL1 in tumor cells via IFNAR1/STAT1 and PD1 in immune cells, resulting in immune suppression in head and neck malignancies (HNSCCs) [122]. IFNAR1 is inactivated in colorectal cancer, altering TME systemic immune responses [123]. Breast tumor spread is accelerated in mice lacking IFNAR1 in NK cells [121].

## 5. T Cells

Studies in cancers have revealed that IFN-I activate CD8^+^ T cells, promoting their expansion [98,99], development and viability [123] and the development of CD8 memory T cells [87,89,90]. In mouse studies, IFN-I and T cell receptor (TCR) signaling play important roles in CD8^+^Ly6C^+^ expansion [124]. The role that IFN-I exert in regulating the expression of co-inhibitory receptors in cancers is under investigation.

The T cell response to IFN-β is controlled by an interactive network that involves two opposing arms of ISG regulation. One arm includes IFN regulatory factors (IRFs) 1/2/4/9 and STATs1-3, while the other arm includes the AP-1 (activator protein-1) transcription factors BATF (basic leucine zipper activating transcription factor-like) and BATF3. SP140 (Speckled Protein 140) was identified as a potential regulator that controls LAG-3 and TIGIT in the two arms, whereas STAT3 is a positive regulator for TIM-3 (T cell immunoglobulin and mucin domain 3). SP140 may have a DNA-binding domain similar to AP-1, which could explain the non-canonical IFN-I signaling in T cells during an IFN-I response [125,126,127,128]. Although SP140 and BCL3 (B-cell lymphoma 3) have no direct links to the traditional JAK-STAT pathway, they can potentially be considered as targets for controlling T-cell co-inhibitory receptors, depending on the context [129]. The deletion of IFNAR1 in mouse cancer cells led to an enhanced anti-tumor response after ionizing radiation, mediated by CD8^+^ T cells. However, malignant cells were more sensitive to CD8^+^ cytotoxicity. It transpires that Serpinb9 (serine protease inhibitor) is an essential factor absent in IFNAR1 knock out cancer cells. Serpinb9, is required for T and NK cell cytotoxicity [130,131,132,133,134]. IFN-I induce Serpinb9 gene expression [135,136,137,138,139]. Targeting Serpinb9 within cancer cells may be beneficial for maximizing IR effectiveness in treating patients with cancer. However, this strategy needs to be carefully considered because Serpinb9 protects cytotoxic effector cells from the destructive effects of granzyme B. Moreover, Serpinb9 is essential for DC-mediated antigen cross-presentation [139,140,141,142,143].

Exposure of tumor cells to IFN-I and IFNγ may have an immunosuppressive effect, mediated by PD-L1 and LGALS9. The persistent IFN signaling in cancer cells can cause epigenetic changes that enhance open chromatin linked with STAT1 and ISGs. Also, the high levels of inhibitory ligands like PDL1 and LGALS9 can cause T cell exhaustion, leading to reduced IFN and CTL function. In tumor cells exposed to IR, there was no evidence of upregulation of PD-L1 [144]. According to Yang et al., treating tumors with anti-IFNβ antibodies leads to enhanced PDL1 blockade, and completer tumor elimination [145]. The presence of PD-L1 and LGALS9 and continuous IFN signaling may be responsible for increased resistance of cancer cells to immune checkpoint blockade, regardless of IR therapy [146].

Notably, CD8^+^ T cells that have an elevated IFN-I gene signature exhibit mitochondrial aberrations, less functional mitochondria, decreased cell viability and are associated with SLE (Systemic lupus erythematosus) development [147], contributing to immune imbalance, inflammation, tissue injury [148] granzyme B release, and the subsequent increase of the autoantigen load [149]. In detail, impaired apoptosis defects can enhance or reduce apoptotic debris clearance, affecting IFN production. Immune complexes may form between self-nucleic acid from apoptotic blebs and autoantibodies, which can stimulate cells to release IFN. In SLE patients, TCR activation and IFN signaling initiate mitochondrial alterations in CD8⁺ T cells, resulting in decreased bioenergetic levels required for T cell function. During an antigen challenge or stress, CD8⁺ T cells stimulated by high levels of IFNα are unable to mount the appropriate response and die, resulting in an increased autoantigen load and subsequent deposition in kidneys causing development/progression to autoimmunity [150].

Elevated IFN concentrations in patients with autoimmune conditions lead to higher levels of activation of effector T cells, which may worsen pre-existing autoimmunity. CD8^+^ T cells play a major role in regulating the generation of autoantibodies, with a CXCR5^+^PD1^+^ T follicular helper subset controlling the autoreactive B cell [151]. These cells function similarly to CD4^+^ T follicular helper cells that promote autoantibody production. Chronic viral infections that result in persistent type I IFN signaling affect the immune response and lead to chronic disorders [152]. IFNα treatment decreases the proliferation of CD8^+^ T cells that recognize disease-unrelated antigens [153] and IFN-I signaling blockade improves virus clearance [154]. IFN-I signaling during persistent LCMV infection in mice promotes immune suppression by inducing the expression of immune checkpoint molecules IL-10 and PD-L1, leading to T-cell exhaustion and lymphoid tissue destruction. However, by blocking IFN-I signaling with a neutralizing antibody against its receptor, this reduced immune system activation, decreased expression of immune checkpoint molecules, resulting in faster viral elimination [154,155]. The impact of chronic type I interferon (IFN) signaling in SLE varies based on the specific immune cell type and environment. The ISG signature in CD4^+^ T cells from SLE patients is associated with a phenotype related to JAK-STAT signaling, T cell co-stimulation and tissue homing [156]. Evidence suggests that persistent activation of IFN signaling is detrimental for CD8^+^ T cells, leading to DNA damage, apoptosis, and activation of the nicotinamide adenine nucleotide (NAD) salvage pathway. T cell metabolism is critical for CD4^+^ and CD8^+^ T cell functional responses [157]. Any metabolic imbalance leads to atypical T-cell function and reduces T cell viability [158]. The IFN signature has been linked to abnormal degradation of mitochondria by autophagy in cells from SLE patients [159,160]. Aberrant mitochondrial metabolism in SLE is predominantly associated with CD4^+^ T cells, and some characteristic defects are related to mitochondrial hyperpolarization, increased mitochondrial size, ATP depletion, and increased production of reactive oxygen species (ROS) [161,162]. TCR stimulation and IFN-I exposure increases CD8^+^ T cell NAD^+^ intake, and impairs mitochondrial metabolism [163]. The data suggest a potential link between IFN exposure and T cell metabolism. In mice, IFNα increases the fatty acid oxidation consumption rate in memory-like OT-1 CD8^+^ T cells. Due to Drp1 (Dynamin-related protein 1) protein defects, SLE patients develop enlargement of mitochondria and increased mitochondrial elongation [164]. NAD^+^ supplementation may help CD8^+^ T cells by restoring mitochondrial function and decreasing mROS buildup in CD8^+^ T cells [165].

## 6. Tregs

Regulatory T cells (Tregs) are critical for limiting an immune response, and providing protection from the development of autoimmunity. However, excessive immune regulation can lead to unrestricted infection and decreased immunity against tumors [166,167,168]. IFN-I play a crucial role in modulating the suppressive functions of regulatory T cells (Tregs) during acute and chronic viral infections [155]. IFNAR-deficient Tregs permit tumor growth and reduce anti-tumor T effector cells, as the lack of IFNAR signaling enhances Treg suppressor function. The rapid production of IFN-I in response to viral infection boosts the persistence of CD8^+^ T cells and reduces Treg-mediated immune suppression. IFNs can decrease the suppressive capacity of Tregs by inducing apoptosis and inhibiting the proliferation of highly suppressive effector Tregs early in infection [169]. This gene expression pattern is observed in both acute and chronic infection models.

A study showed that LCMV clonel-13 infection led to prolonged elevated virus levels in serum, kidneys, and lungs for up to 46 days, along with decreased effector cytokine production and CD8^+^ T lymphocytes specific to antiviral antigens. Enhanced viral persistence resulted in upregulation of markers associated with T cell exhaustion [170,171,172] and reduced production of antigen-specific memory CD8^+^ T cells. IL-10-generating Treg cells were found to enhance memory T cell generation after LCMV Armstrong infection, contrasting with decreased memory T cell production in clone-13 infection [173]. The study also highlighted the role of genes Erdr1, Rell1, and Tlr7 in immune suppression by Tregs during IFNAR signaling in LCMV infection [174,175,176]. IL-21 signaling affects Treg activity in LCMV disease, resulting in prolonged viral presence [177]. Additionally, LCMV infection induced a loss of Treg cells compensated by Vβ5+ conventional T cells converting into iTreg cells, impacting the antiviral immune response, priming CD8 effector T cells and leading to colitis [178].

Gangaplara et al. studied the impact of IFNAR signaling in Tregs during infection and in cancer mouse models. They found that the absence of IFNAR signaling in cancer models led to increased tumor growth and reduced efficacy of antitumor T effector cells [179]. This was associated with an increase in activated PD-1^+^ tumor-infiltrating Tregs and suppression of CD4^+^Foxp3^−^ and CD8^+^ T cells in tumor tissues. The activation of Tregs in the TME involved both direct and indirect type I IFN signaling in Tregs and non-T cells. Type I IFN signaling helped maintain stable STAT1 levels and activate tumor regulatory T cells. Additionally, Treg IL-10 production regulated the Th17 response in the TME [180], with TME-specific type I IFN signaling promoting Treg activation and IL-10 synthesis to counteract Th17 inflammation and prevent tumor development or autoimmunity [181,182,183]. Tregs exhibit selective activation in response to both self and non-self-antigens, as well as type I IFN signaling [184].

As described, IFN signaling plays a crucial role in regulating the immune response. IFN-I activate Tregs in the TME, maintaining STAT1 levels. IFNγ activates T-bet in Th1 CD4^+^ T cells through STAT1, creating a positive feedback loop with IL-12 and IFNγ [23,185,186]. IFN-I activate STAT3 to enhance granzyme B expression in cytotoxic T cells, boosting their effector function [187]. A computational model showed that IFNβ enhances Tregs via STAT1- and P300-dependent Foxp3 acetylation [188]. IL-2 and STAT5 are essential for Treg homeostasis and function [189,190]. Mst1 amplifies IL-2-STAT5 activity in Tregs, crucial for preventing tumor resistance and autoimmunity. STAT5 prolongs Treg Foxp3 production, while STAT3 promotes Th17 cell differentiation [190]. Overall, IFN signaling plays a complex role in immune regulation and cell function.

Treatment with TLR7 or TLR9 agonists reduces Treg in mouse tumors by IFN-I activity, enhancing CD8^+^ T cell activity through DC activation and intra-tumoral Treg suppression [191,192]. Notably, myeloid and tumor cells expressing PD-L1 and IDO influence Tregs’ ability to suppress T-cell activity [102,193]. IFN-I can prompt Tregs to secrete IL-10 in the tumor microenvironment (TME), enhancing their suppressive capability. The TME plays a crucial role [194].

## 7. Macrophages and Myeloid-Derived Suppressor Cells (MDSC)

MDSC, tumor-associated macrophages (TAMs) and tumor-associated neutrophils (TANs) constitute approximately 50% of the hematopoietic cell population in the TME [195,196,197,198]. TAMs and TANs are responsible for the progression to malignancy, generating immunosuppressive cytokines, reactive oxygen species (ROS), reactive nitrogen species (RNS) and angiogenic factors [199,200,201,202] that cause persistent inflammation within tumors and inhibit the immune response [203]. IRF4, associated with the M2 macrophage type, is characterized by its suppressive properties that facilitate tumor progression [204,205].

Obesity can lead to compromised immune responses in respiratory epithelial cells and macrophages, resulting in elevated levels of inflammatory cytokines and increased susceptibility to severe outcomes, e.g., during epidemic influenza infections [206,207,208]. Reduced IFN production and compromised ISG levels may constitute a potential risk factor for individuals who are obese [209,210]. Obesity affects the immune system by increasing SOCS3 (Suppressor of cytokine signaling) and altering leptin levels, leading to impaired IFN-I responses after TLR activation, and dysfunction of both T and B cells [211,212,213,214]. Studies infer a reciprocal relationship between IFN-I and metabolism [32,33]. Cancer cells can produce DAMPs which contribute to the modification of the tumor immune TME through the activation of different PPRs presented on immune cells. Tumor cells utilize various metabolic intermediates—ATP-binding cassette (ABC), lactate, glucose, and amino acid transporters—to enhance their viability, spread, and tissue invasion [215,216]. By activating STING, GAMP levels in the TME reduce the number of MDSCs and promote IFNγ secretion from cytotoxic antitumor effector cells, suppressing cancer metastasis [217]. In the context of MDSCs, cGAMP could prevent the production of ROS and RNS, thereby impeding the immunosuppressive TME. ABCC1 protein is important for the efflux of cyclic GMP-AMP (cGAMP), facilitating its subsequent uptake by immune cells, (MDSCs and macrophages) [218].

The activation of STING and subsequent production of IFN-β by TME cGAMP is limited to myeloid cells and B cells, excluding NK cells [79]. When exposed to tumor-derived cyclic GMP-AMP (cGAMP), myeloid cells produce IFN-I which subsequently activate NK cells to exert cytotoxic effects against tumors [219]. The immunosuppressive capacity of MDSCs is affected by STING, which enhances T cell expansion and hinders the differentiation of MDSCs [220,221]. The antitumor effects of ICIs (immune-checkpoint inhibitors) are determined by the cGAS/STING signaling axis on myeloid immune cells. A lack of PD-L1 leads to the downregulation of molecules associated with DNA repair, thereby facilitating the uptake of DNA from apoptotic tumor cells by myeloid cells to stimulate the cGAS/STING axis [222]. The inhibition of PD-L1 in human DCs augments the antitumor efficacy of CD8^+^ T-cells against tumor cells expressing PD-L1 in a cGAS-dependent manner [223,224].

Consequently, active/STING signaling is necessary for myeloid immune cell-mediated tumor immunity in the TME. The role of transmembrane protein 203 (TMEM203) in macrophages from diverse solid malignancies is significant, given its interaction with STING and its involvement in modulating pro-inflammatory signaling upon detection of cGAMP [225]. The production of STING-dependent IFN-I through the focal adhesion kinase (FAK)/sirtuin-3 (SIRT3)/ROS axis is triggered by CD11b stimulation on TAMs. Mitochondrial DNA activates the cGAS/STING signaling pathway, activating STAT1-dependent antitumor immune responses [226,227,228]. In vivo, TLR ligands induce immune activation, producing multiple proinflammatory cytokines [229]. Nevertheless, the activation of TLR7 and TLR9 predominantly lead to the production of significant amounts of IFN-α by plasmacytoid DCs (pDCs) [45,230]. Neovascularization (angiogenesis and vasculogenesis) is initiated is by VEGF secretion from malignant cells and TAMs, [231,232]; IFN-I possess significant potential in preventing angiogenesis and neovascularization [233,234].

## 8. Neutrophils, TANs, and G-MDSC

The inflammatory cytokines IL-1 and IL-6 and PGE2 in the TME affect the development and immunosuppressive properties of MDSC [235]. Both MDSC and G-MDSC populate the TME, with G-MDSC generally being the larger population (70–80%) [236,237,238]. Tumor associated neutrophils (TANs) are characterized as N1 (cytotoxic and anti-tumorigenic properties) or N2 (phenotype associated with tumor progression) [239,240]. IFN-β promotes N1 activity whereas TGF-ß (transforming growth factor-ß) is linked to N2 differentiation [239,241,242]. TANs are important players shaping tumor immunity and cancer progression [243]. TANs and G-MDSCs immune modulatory properties [244]. In the context of IFN-I signaling, the PI3K-Akt/mTOR pathway is regulated by SOCS1 [225]. Reduced levels of IFN-I in the TME results in enhanced PI3K-Akt-mTOR signaling in G-MDSCs, thereby permitting tumor growth, suggestive for a role of IFN-I in limiting G-MDSC activity.

IFN-I are also implicated in neutrophil maturation and the establishment of immune functionality [245].

**Figure 4:**
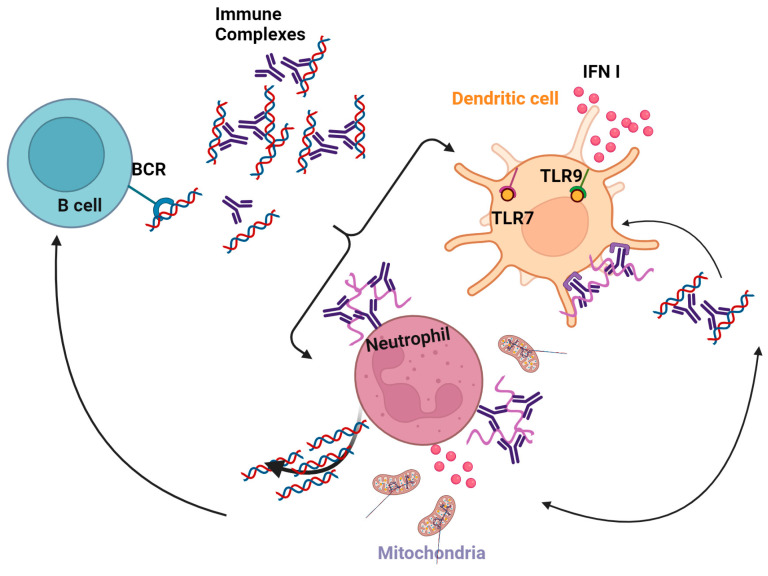
Neutrophils and DCs contribute to autoimmunity. Activated neutrophils release IFN-I and are involved in B cell activation in SLE. Created with Created https://BioRender.com, accessed on 26 November 2023.

Mature neutrophils exhibit a pronounced upregulation of specific genes that enhance their responsiveness to IFN-I and IFNγ. Both IFNα and IFNγ elicit robust tyrosine phosphorylation of STAT1 in mature neutrophils, while no such effect is observed in immature neutrophils [58]. IFNs prime mature neutrophils, increasing their ability to produce extracellular traps (NETs), associated with TLR activation [246] and complement factor 5a (C5a) [245]. Neutrophils in SLE stimulate DCs by releasing immune complexes containing nucleic acids and anti-DNA autoantibodies SLE [247] (Figure 4). The presence of IL-37 in SLE immune complexes leads to the stimulation of TLR9 in pDCs, resulting in the subsequent synthesis of IFNα [248].

## 9. B Cells

Antibody producing B cells have a critical role in both viral clearance and antitumor activity. IFN-I enhance B cell activation by increasing the expression of co-stimulatory molecules, leading to more robust B cell activation [92,123,249]. IFN-I contribute to the formation of antibodies both in vitro and in vivo by facilitating the process of isotype switching, leading to, for example, the production of IgG2a/c antibodies [250,251,252,253]. Mice lacking IFNAR on their B cells exhibit a diminished ability to mount an antibody response and isotype switch, compared to mice with B cells expressing wild-type IFNAR [254].

Previous studies have established that the activation of IFNAR by B cells is not critical in developing antiviral IgG2c responses during severe influenza infection [252]. The enhanced response of follicular B cells to elevate CD69 expression when exposed to IFNα and BCR-mediated stimulation likely ensures that these normally circulating B cells stay in lymphoid tissues, allowing them to acquire supplementary indirect secondary signals that are important for the development of T cell-independent-2 antibody feedback [255,256].

Activated macrophages and DCs produce IFN-I and IL-12 that contribute to NK cell activation. In turn, activated NK cells produce IFNγ that enhances the level of B cell synthesis of IgG2a [257,258]. IFNγ also influences B cell function depending on the activation or differentiation state of the B cells [259]. Since activated B cells can induce tumor cell death and IFNγ [117,118,260,261], B-cell vaccines are being examined for their therapeutic potential. Activated NK cells can initiate the necessary processes for switch recombination in B cells stimulated by antigens, by activating the transcription of downstream exons in the germline [43,262]. The interaction between CD2 or CD244 receptors on NK cells and CD48 receptors on B cells is, however, not cytokine dependent.

## 10. Conclusions

IFN-I have a pivotal role in controlling tumor proliferation and modulating anti-cancer immunity. Studies have revealed that immune evasion and subsequent tumor progression is, under certain circumstances, facilitated by the early loss of IFN-I signaling. A robust and distinct IFN-induced ISG signature is associated with both direct tumor cell death and immune cell-mediated tumor clearance. By contrast, low, sustained levels of IFN-I, present in the TME, lead to a distinct ISG signature that may be associated with tumor progression.

**Figure 5:**
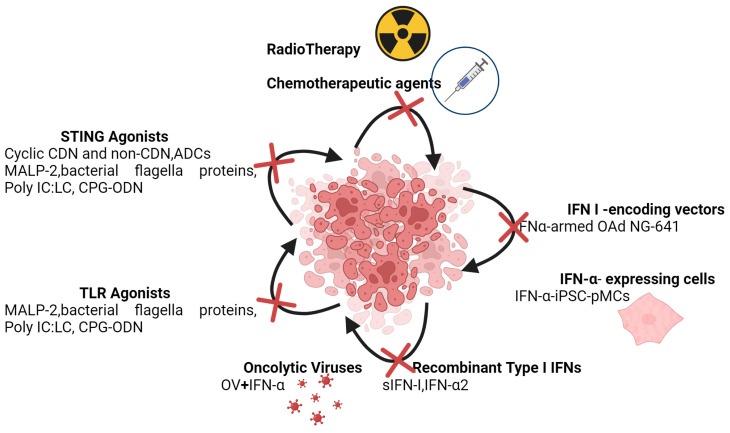
IFN-dependent immunotherapies. IFN-I are vital in modulating the immune response within the TME and contribute the effectiveness of different immune therapies. Created with https://BioRender.com, accessed on 26 November 2023.

As outlined in the preceding, the challenge is to discern the context in which to consider IFN-I as anti-cancer therapeutic agents (Figure 5).
